# Probiotic *Lactobacillus fermentum* strain JDFM216 improves cognitive behavior and modulates immune response with gut microbiota

**DOI:** 10.1038/s41598-020-77587-w

**Published:** 2020-12-10

**Authors:** Mi Ri Park, Minhye Shin, Daye Mun, Seong-Yeop Jeong, Do-Youn Jeong, Minho Song, Gwangpyo Ko, Tatsuya Unno, Younghoon Kim, Sangnam Oh

**Affiliations:** 1 Department of Animal Science and Institute of Milk Genomics, Jeonbuk National University, Jeonju, 54896 South Korea; 2 Department of Agricultural Biotechnology and Research Institute of Agriculture and Life Science, Seoul National University, Seoul, 08826 South Korea; 3 Microbial Institute for Fermentation Industry, Sunchang, Jeonbuk 56048 South Korea; 4 Division of Animal and Dairy Science, Chungnam National University, Daejeon, 34134 South Korea; 5 Subtropical/Tropical Organism Gene Bank, Jeju National University, Jeju, 63243 South Korea; 6 Faculty of Biotechnology, Jeju National University, Jeju, 63243 South Korea; 7 Department of Functional Food and Biotechnology, Jeonju University, Jeonju, 55069 South Korea

**Keywords:** Microbiology, Health care

## Abstract

Increasing evidence indicates that alterations in gut microbiota are associated with mammalian development and physiology. The gut microbiota has been proposed as an essential player in metabolic diseases including brain health. This study aimed to determine the impact of probiotics on degenerative changes in the gut microbiota and cognitive behavior. Assessment of various behavioral and physiological functions was performed using Y-maze tests, wheel running tests, accelerated rotarod tests, balance beam tests, and forced swimming tests (FSTs), using adult mice after 50 weeks of administering living probiotic bacterium *Lactobacillus fermentum* strain JDFM216 or a vehicle. Immunomodulatory function was investigated using immune organs, immune cells and immune molecules in the mice, and gut microbiota was also evaluated in their feces. Notably, the *L. fermentum* JDFM216-treated group showed significantly better performance in the behavior tests (P < 0.05) as well as improved phagocytic activity of macrophages, enhanced sIgA production, and stimulated immune cells (P < 0.05). In aged mice, we observed decreases in species belonging to the Porphyromonadaceae family and the *Lactobacillus* genus when compared to young mice. While administering the supplementation of *L. fermentum* JDFM216 to aged mice did not shift the whole gut microbiota, the abundance of *Lactobacillus* species was significantly increased (P < 0.05). Our findings suggested that *L. fermentum* JDFM216 also provided beneficial effects on the regulation of immune responses, which has promising implications for functional foods. Taken together, *L. fermentum* JDFM216 could confer the benefit of improving health with enhanced cognition, physiological behavior, and immunity by modulating the gut microbiota.

## Introduction

The prevalence of aging-associated diseases has been increased according to the arising of elderly population, and functional foods that furnish health-promoting effects to control aging and longevity including health span have become more desirable. Physiological changes related with aging include impaired memorial and cognitive functions, suppressed immune responses, and dysbiosis of bone remodeling^1^. Importantly, environments in early life could remarkably influence on later development, maturation, and functionality of the host. Especially, gut microbiota co-exists in a mostly symbiotic partnership with its host during the entire life cycle. At birth, colonization by the microbiota participates in maturation of intestinal barrier function and gut homeostasis as well as immune response including innate and host adaptive immunity^2^. The microbiota undergoes dramatic changes in richness and composition during adulthood, contributing to cellular homeostasis and disease onset in later life^3^.

The dynamics of gut microbiota have been increasingly recognized to modulate host physiology and are linked to central nervous system function in human and animal studies^4,5^. Importantly, emerging evidences propose that a gut–brain axis exists and that it plays an important role in modulating cognitive functions and behavior^6^. Many studies have shown the microbiota and its role in neurological disorders such as multiple sclerosis, autism, Parkinson’s disease, and Alzheimer’s disease^7–11^. And supplementation of probiotics is highly associated with neuroprotective effects in hosts with the brain disorders. Therefore, the modulation of gut microbiota may be new strategy for cognitive disorders related to neurodegenerative diseases and depression as targeting new pathway.

Probiotics, live microorganisms that provide health benefit to the host when administered in adequate amounts, reveal on strong interaction with gut commensals. Among them, it has been well-established that therapeutic interventions of lactobacilli improve cognitive behaviors for mitigating mood disorders as well as alters gut microbiota composition^12–14^. Moreover, supplementation with probiotics restores harmful effects observed on the immune and cognitive functions due to aging through anti-inflammation and anti-oxidative stress^15^. Among the species of *Lactobacillus*, it has been reported that *Lactobacillus fermentum* confers symbiotic impacts by stimulation of effective immunity and antioxidant activity in aging mice^16^. Similarly, in our previous studies, we showed that one strain of *Lactobacillu*s species, *L. fermentum* JDFM216, exerts probiotic properties such as adhesive properties, lifespan extension, strengthening of the immune system and other health-promoting functions in a *Caenorhabditis elegans* animal model^17^. Here we focused on understanding whether and how microbiota alterations by supplementation with *L. fermentum* JDFM216 contribute to behavior and immune responses, and whether physical and mental decline could be targeted by gut microbiota modulations using the aged mouse model.

## Results

### JDFM216 affected the behaviors of aged mouse

In the current study, our research objective was to understand how probiotic supplementation contributes to behavior and immune responses in the aged mouse model, since a number of human clinical trials have been reported on the effects of long-term probiotics supplementation in middle-aged and older adults to examine their impact on cognitive functions^18–21^. Therefore, we employed 12-month-old (52 weeks) mice as a model of middle-aged mice according to the relation between mice age and human age^22^, and JDFM216 was administered for 48 weeks to ensure that senescence was sufficiently developed.

**Figure 1:**
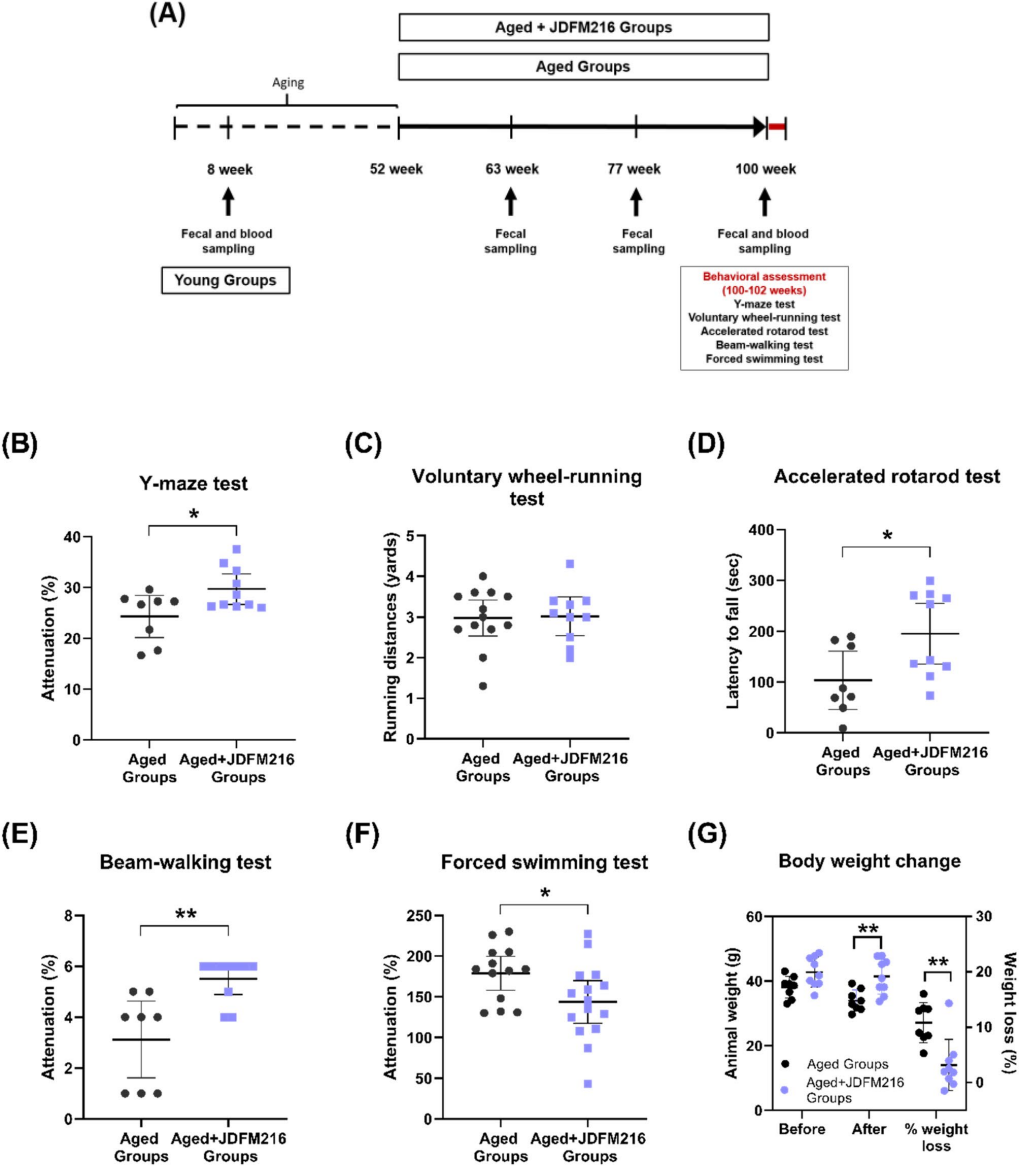
Behavioral assessments of JDFM216-treated aged mice. (**A**) Representation of the study design. JDFM216 or vehicle (sterile water) was administered for a total period of 48 weeks to C57BL/6 mice. (**B**) Y-maze test. (**C**) Voluntary wheel-running test. (**D**) Accelerated rotarod test. (**E**) Beam-walking test. (**F**) Forced swimming test (FST). (**G**) The average weight and % weight loss of aged mice before and after the battery of behavioral tests (100 and 102 weeks). For all graphs, data are expressed as the means ± SD. n = 13–15 per group. Levels of significance determined by the Student’s *t* test are as follows: *P < 0.05; **P < 0.01; ***P < 0.001, compared with the aged mouse group.

This study first aimed to assess the effects of JDFM216 on age-related cognition, memory impairment and brain functions. In the Y-maze test, oral administration of JDFM216 significantly increased spontaneous alternation by 4.46% compared with the control group (Fig. 1B; *P* = 0.0288). All aged mice with JDFM216 treatment significantly alternated between arms above chance level at 22.2%, while the performance of the control group was not different from the level. Since a higher percentage of alternations has been associated with better recognition and thus increased memory, these data implicate that JDFM216 may improve spatial working memory in aged mice. It is noted that although the Y-maze test is not the most specific test for memory, Y-maze function is sensitive to damage in areas such as the hippocampus highly associated with cognition and neurodegenerative diseases^23^. Also, these data are consistent with prior observations that *L. plantarum* C29 strain ameliorates memory impairment caused by aging^24^, implying the probable efficacy of JDFM216 for improvement of memory. We next conducted the wheel-running test for voluntary exercises to determine locomotor activities. Spontaneous running activities were not significantly different between the control and JDFM216-treated groups, and mice ran an average of approximately 3.0 yards for 5 min in both groups (Fig. 1C). These findings indicate that dietary supplementation with JDFM216 has no effect on the capacity for voluntary exercise. Motor learning abilities were also measured by the accelerating rotarod test. This test is widely used to evaluate the motor coordination of rodents and is especially sensitive for detecting cerebellar dysfunction. In this test, motor performances of mice were analyzed by measuring the latency to falling off a rod that was rotated with increasing velocity. As a result, the JDFM216-treated group showed a significantly increased latency to fall by 88.6% compared with the control group (Fig. 1D; *P* = 0.0234), suggesting that JDFM216 enhances motor performance in aged mice. Sense of balance and movement function of the back foot of the aged mice were further assessed using the balance beam-walking test, which analyses the number of foot slips. Mice were trained to traverse a medium (40 mm wide) square beam for 2 days, and the number of hind paw slips was scored and video-recorded on the test days on a narrow (30 mm wide) beam. As a result of the test, JDFM216-treated mice showed significantly higher scores with fewer slips by 47.9% compared with control mice (Fig. 1E; *P* = 0.0046), suggesting better motor balance ability. It is worth mentioning that mice are known to lose their visual acuity with age^25^. The effect of JDFM216 on the improved motor balance could result from alleviated loss of the visual function in aged mice by the JDFM216 administration, and further investigation is required to specify the mechanism.

Currently, depression is one of the most common psychiatric disorders and has become a public health concern, since it is associated with high morbidity and mortality. Thus, we finally performed the forced swimming test (FST), which is the most widely used behavioral test to measure the level of depression of drugs and compounds. Notably, this test demonstrated that JDFM216 exhibited an antidepressant-like effect by showing a significantly decreased immobility time by 19.6% compared with the control group (Fig. 1F; *P* = 0.0353). We also weighed all the mice biweekly during the whole period of studies, and there were no significant differences in body weight gain between the groups that received the JDFM216 treatment and the control up to beginning the behavioral tests. Importantly, the mean percentage of weight loss changed significantly between the two groups before (at 100 weeks) and after (at 102 weeks) the behavioral assessments (Fig. 1G, P = 0.0018). Aged mice in the control group lost, on average, approximately 10.8% of their body weight after the overall behavior tests, and those in the JDFM216-treated group showed a 3.1% weight loss (approximately 7.4 ± 0.2 g lighter in the control mice than those that received treatment). We speculate that the difference may be because mice in the control group were more vulnerable to stress. Altogether, these results indicate that the JDFM216 might have an effect in aged mice manifesting as resistance to environmental stresses from continuous test-related behavior, suggesting that the probiotic administration of JDFM216 can help to improve the brain functions during the old age (Supplementary movie).

### JDFM216 modulated the immune system of aged mouse

**Figure 2:**
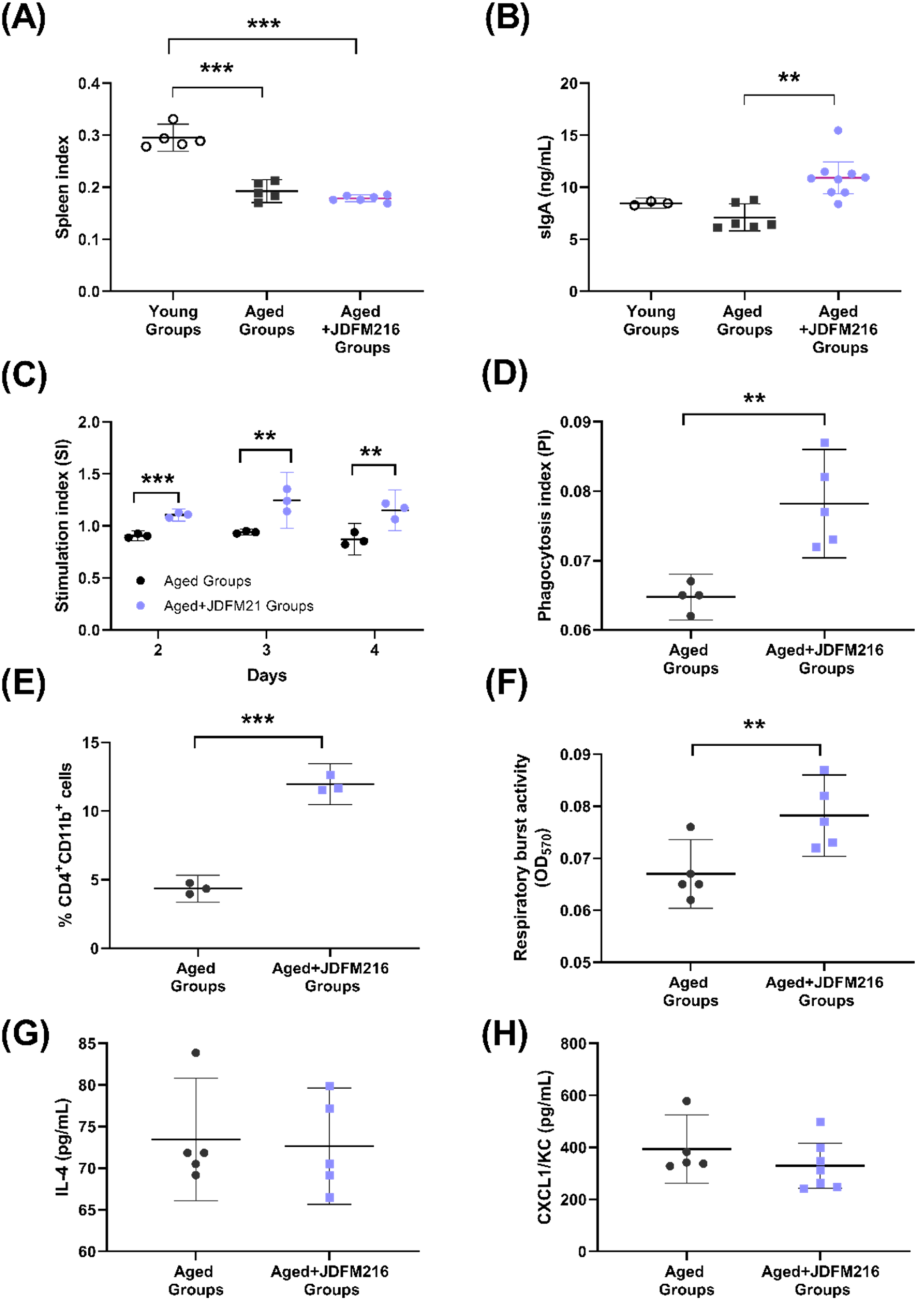
Profiling of the immune response in JDFM216-treated aged mice. (**A**) Spleen indices of young and aged mice (Young Groups, Aged Groups or Aged + JDFM216 Groups). (**B**) Secretary immunoglobulin A (sIgA) production in the intestine. (**C**) Proliferation of splenocytes. The proliferation was measured at 48, 72, and 96 h of cultivation. (**D**) Pinocytosis of peritoneal macrophages. (**E**) CD11b and CD4 expression on the surface of dendritic cells. (**F**) Leukocyte respiratory burst activity. Cytokine IL-4 and chemokine CXCL1 levels. (**G**,**H**). The young mouse group was used at 8 weeks of age. Values are means ± SD. Asterisks represent statistically significant differences; one-way ANOVA was performed. *P < 0.05; **P < 0.01; ***P < 0.001, compared with the groups.

After the behavioral assessments, all mice were sacrificed to determine the immunoregulatory effects. Spleen samples were collected first; this observation was of interest as the mammalian spleen size has been correlated with differences in immune cell subsets and resistance to infection by encapsulated bacteria^26^. In our studies, compared with the Young Groups (8-week-old mice), the spleen indices in both of two aged mice groups (Aged Groups and Aged + JDFM216 Groups) showed a significant decrease (Fig. 2A, P < 0.0001), although there was no significant difference depending on the JDFM216 treatment. Next, the intestinal content of each mouse was collected from small intestine sections, and the level of secretory immunoglobulin A (sIgA) production was determined. sIgA is the main immunoglobulin found in mucous secretions that plays a critical role in mucosal immunity protecting vulnerable areas such as the gut from invading pathogens^27^. In accordance with previous reports showing an increased level of sIgA by lactobacilli administration^28,29^, JDFM216 stimulated aged mice to produce a significantly high level of sIgA (over 10 ng/mL; *P* = 0.0015) compared with the control group (Fig. 2B). Lymphocyte proliferation is commonly examined to assess the cell-mediated immunity when analyzing the efficacy of vaccines, diagnosis of infectious disease and immune deficiencies^30^. Approximately 5.0 × 10^6^ splenocytes were separated from mice, and the proliferative capacity of splenic T-lymphocytes was measured. As a result, the JDFM216-treated group showed a significant increase in stimulation index (*SI*) values over 4 days compared with the concanavalin A (ConA) control group (Fig. 2C; P < 0.05). These results indicate that JDFM216 can stimulate a T-lymphocyte proliferative response. To examine the phagocytic activity of peritoneal macrophages, quantitative spectrophotometric determination of neutral red accumulation in cells was measured in each group. It has been shown that stress stimulates the phagocytic capacity of macrophages preventing the entrance and survival of microorganisms in situation of immunodepression^31^. Figure 2D shows that the phagocytosis index (*PI*) value in JDFM216-treated group was increased by nearly 30% compared with the control group (*P* = 0.0041). Dendritic cells play a pivotal immunoregulatory role with priming of the T helper cells, and probiotic bacteria are involved in modulation of the dendritic cell phenotype and function^32–34^. Splenocytes were separated from mice and used to investigate the effect of JDFM216 on CD11b and CD4 expression on the surface of dendritic cells by flow cytometry. Specifically, the percentage of CD11b^+^CD4^+^ cells in the JDFM216-treated group increased to 7.6%, which was 2.7-fold higher than the PBS control group (Fig. 2E; P < 0.001), indicating that JDFM216 has the ability to stimulate the maturation of myeloid dendritic cells. The respiratory burst assay also showed that JDFM216 could activate mouse innate immunity, as seen in Fig. 2F. The leukocyte respiratory burst activity significantly increased in the JDFM216-treated group (*P* = 0.016), indicating that phagocytes enhanced the production of ROS by activating the NADPH oxidase enzyme that produces oxygen peroxide. In addition, we examined cytokines chemokine markers including IL-4, leukocyte recruitment (*CXCL1*), IL-10, IL-6 and TNF-alpha in mice serum (Fig. 2G,H and Fig. S1). These markers showed no significant differences, although a slight decrease was observed for IL-4 and *CXCL1* with JDFM216 when compared with the controls. We also evaluated the cortisol level, but there was no significant difference between the control and JDFM216-supplemented groups (Fig. S1A). It is worth noting that the immunological measures were conducted after the behavioral tests which could be a potential acute stress to the mice, although the two groups of mice were treated in the same manner. The acute stress tends to induce leukocyte redistribution of cells and alter the innate immune responses, so the feasible confounding by the stress on the effects of immune responses could not be ruled out^35^.

### Feeding JDFM216 influences the diversity of the gut microbiota in aged mice

**Figure 3:**
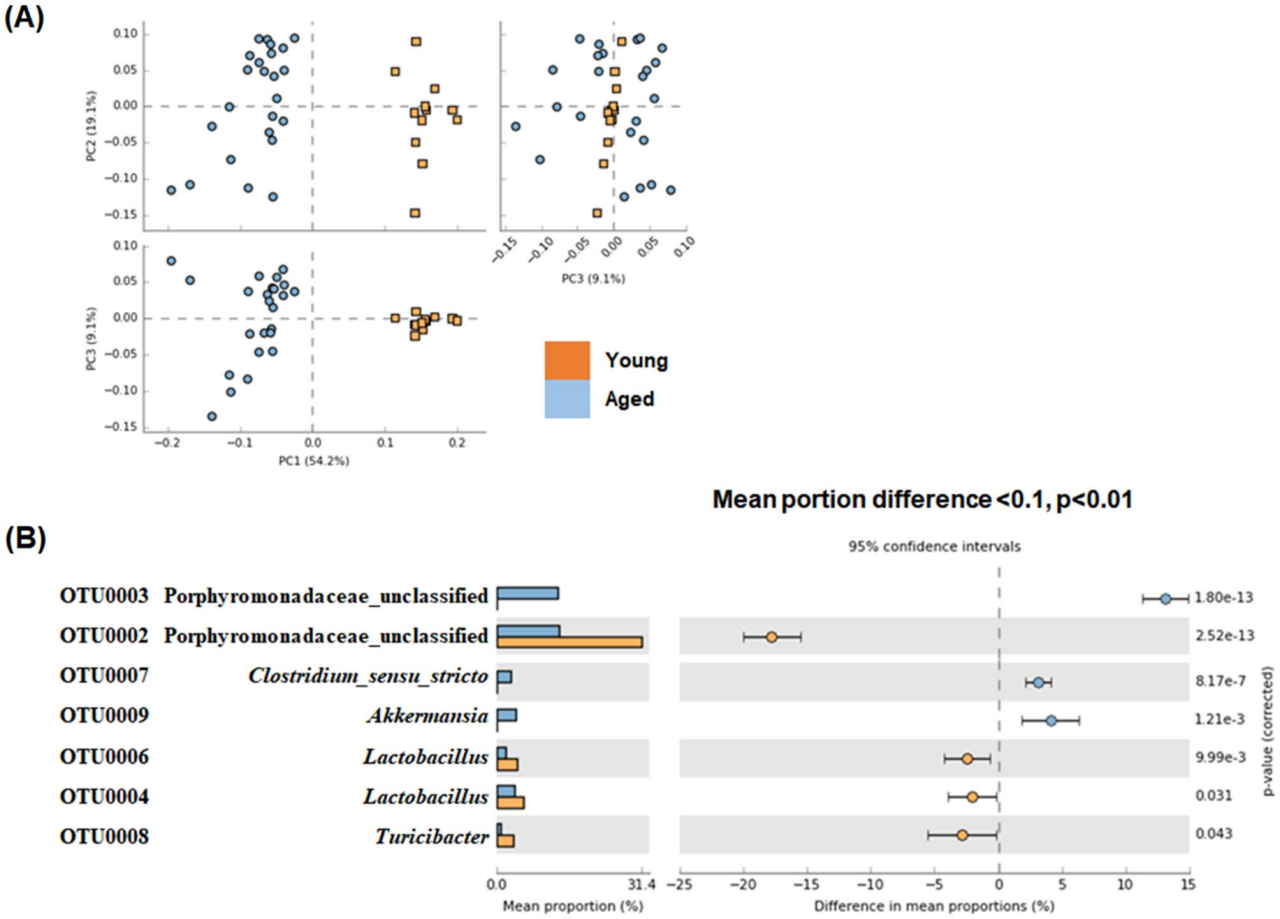
Comparison of gut microbiota between young mice (Young Groups at 8 weeks) and aged mice (Aged Groups at 100 weeks) based on principal component analysis (**A**) and operational taxonomic units (OTUs) with significantly different abundance (**B**). Corrected *P* values are shown on the right. The effect sizes and confidence intervals are provided to allow for critical assessment of the biological relevancy of the test results.

To determine the impact of JDFM216 on the mouse gut microbiota, we analyzed the species richness and evenness. After normalizing the number of reads per sample, we obtained a total of 29,122 unique high-quality reads (14,000 reads per sample) that showed more than 99% Good’s coverage for all samples (data not shown). First, comparison of the ecological indices showed that species richness (Chao index) and evenness (Shannon index) were higher in aged mice microbiota regardless of the JDFM216 treatment (Fig. S2), confirming the previous findings on increased diversity of gut microbiota with age in human and mice^36–38^. Results from Fig. 3A suggest that the aged mouse microbiota was significantly different from the young mouse microbiota (P < 0.001). The differential abundance test indicated that OTUs belonging to the family Porphyromonadaceae and the genus *Lactobacillus* and *Turicibacter* were more abundant in young mice and that other OTUs belonging to the family Porphyromonadaceae and the genus *Lactobacillus*, *Clostridium *sensu stricto and *Akkermansia* were more abundant in old mice. The mean portion difference was highest for the Porphyromonadaceae OTU (Fig. 3B). *Akkermansia muciniphila* is a well-known mucin-degrading bacterium that is associated with anti-inflammatory effects in human. Similarly to our results, the bacterial composition tended to increase by aging possibly along with normal mucosa development^39^.

**Figure 4:**
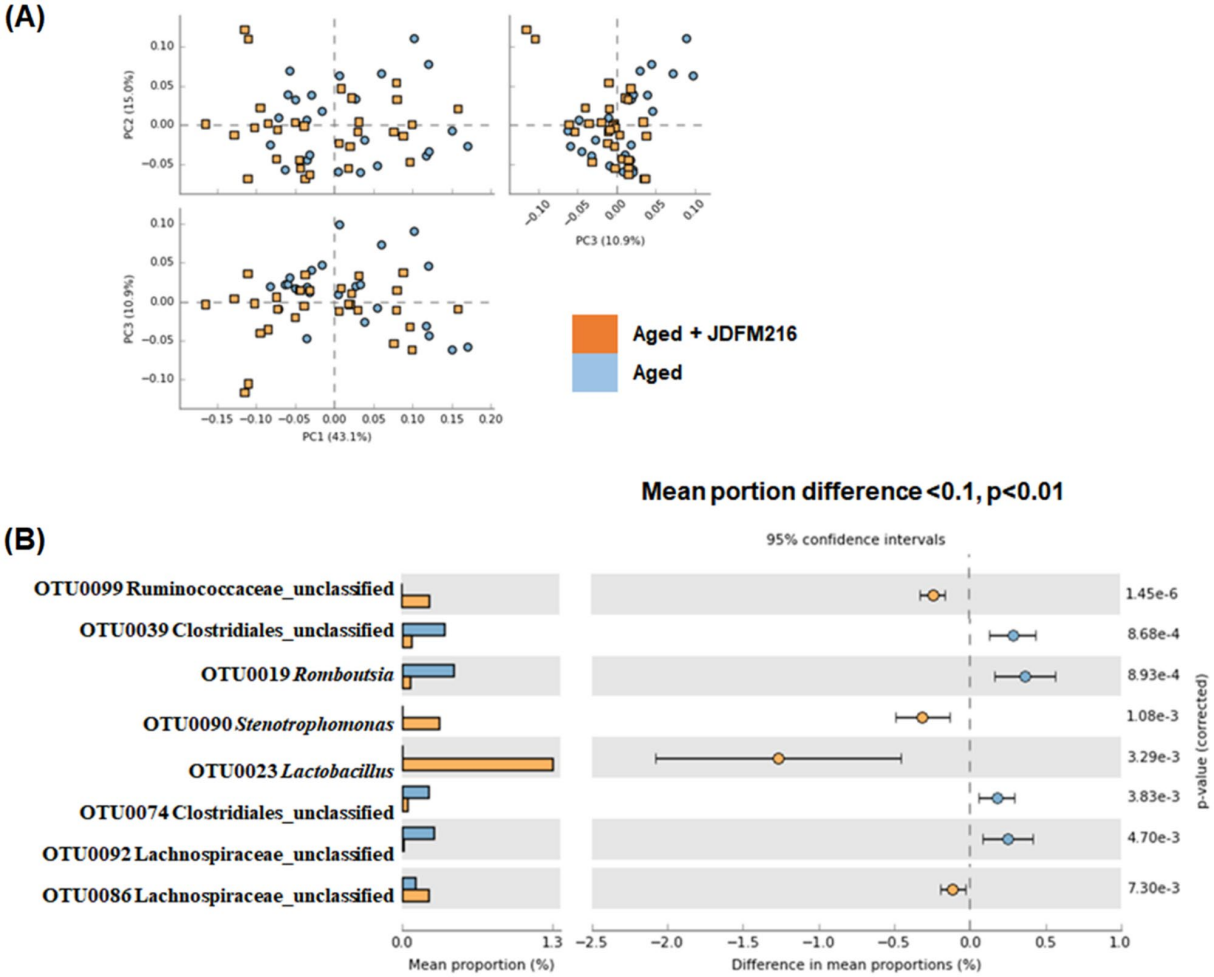
Comparison of gut microbiota between aged mice (Aged Groups) and aged mice fed *Lactobacillus fermentum* JDFM216 (Aged Groups + JDFM216) at 100 weeks based on principal component analysis (**A**) and operational taxonomic units (OTUs) with significantly different abundance (**B**). Corrected *P* values are shown on the right. The effect sizes and confidence intervals are provided to allow for critical assessment of the biological relevance of the test results. Significantly increased (**A**) and decreased OTUs (**B**) and by feeding JDFM216.

Among aged mice, we did not observe a significant shift in the microbiota by feeding JDFM 216 (Fig. 4A), but 4 and 7 OTUs were significantly increased and decreased by feeding JDFM216, respectively. Among them, OTU0023 *Lactobacillus* was increased by far the most, followed by OTU0090 *Stenotrophomonas* and OTU0099 Ruminococcaceae unclassified. The abundances of other OTUs belonging to the family Lachnospiraceae and Ruminococcaceae and the genus *Ruminococcus* were lower in the mice fed with JDFM216 (Fig. 4B). *Stenotrophomonas* is a genus of Gram-negative bacteria, comprising a broad range of species from beneficial probiotics (*S. acidaminiphila*) to opportunistic human pathogens (*S. maltophilia*)^40,41^. In the view of gut microbiota, there is a report showing the enriched *Stenotrophomonas* in the *Lactobacillus plantarum*-treated group similar to our finding^42,43^. It is noted that the current 16S metagenomics sequencing result indicates high abundance of *Lactobacillus*, probably present in the recipient intestines. In our previous study, we have shown that JDFM216 exhibits significantly high colonization ability to the intestinal tract of *C. elegans*^44^. Also, *L. fermentum* is known to possess an excellent adhesion ability in mice^45,46^. In view of the findings, we speculate that JDFM216 would colonize the intestinal tract of the murine mouse model.

Next, we explored the metabolic activity of the gut microbiota in aged mice through PICRUSt analysis to predict the functional profiling of the microbial communities based on the 16S rRNA gene sequences. Interestingly, the mice in the aged groups showed a significant increase in the metabolic pathways of butanoate and propionate compared with the young groups (Fig. S3). Conversely, comparison of the metabolic activity between the aged and aged + JDFM216 groups did not show a dramatic difference (Fig. S4). The OTUs that showed a higher abundance among JDFM216-treated mice were further investigated to evaluate the association with metabolic activities estimated by PICRUSt2. These OTUs showed positive and negative moderate correlation (− 0.5 < correlation < 0.5, p < 0.05) to 583 and 48 KEGG enzymes, respectively. Among them, the KEGG enzymes with a moderate correlation to the abundance of OTU0023 *Lactobacillus* are summarized in Table S1 and S2. Importantly, KEGG enzymes that were increased by feeding JDFM216 included vitamin B1, B2, and B6, butanoate, lipoic acid and taurine metabolism, all of which have been implicated in anti-aging effects. On the other hand, KEGG enzymes with a reduced abundance in the treated group included antibiotic metabolism. To support the functional profile prediction of OTU0023 *Lactobacillus*, we analyzed metabolites production of JDFM216 in the bacterial culture supernatant. As shown in Fig. S5, we could detect metabolic derivatives of butanoate including methyl-malonate, butanedioic acid, and butane-2,3-diol, of which abundances increased in the culture supernatant of JDFM216 compared to the blank medium. This suggests that JDFM216 supplementation would supply butanoate to the host.

## Discussion

Aging accompanies severe chronic diseases that requires further investigation of mitigation approaches. There is now mounting evidence pointing to an important link among microbiota, behavior and the immune system^47^. Here we aimed to explore the impact of oral administration of JDFM216 on cognitive behaviors and immune responses in an aged murine model. We therefore designed intervention studies of JDFM216 for healthful longevity. *L. fermentum* JDFM216 has been studied for its health-promoting activities of longevity and immune response of the host, possessing phylogenetically featured genes related to the activities^44,48^.

As shown in the present study, JDFM216 induced a significant positive modulation of cognitive behaviors and physiological properties in aged mice. This result was supported by higher performances on Y-maze, accelerated rotarod, beam-walking and forced swimming tests with the administration of JDFM216. Other investigations have reported the effect of probiotics for improving memory in middle-aged rats as a therapeutic agent using the Morris water maze and Pavlovian autoshaping tests^49^. The anti-depressant effect of probiotics has been demonstrated in animal models by the FST and tail suspension test (TST)^50,51^. In addition to these reports, the cognitive functions being altered by probiotics are mostly psychiatric disorders and memory abilities, which were confirmed in the current study^52^. Our results of the JDFM216 treatment also suggested improvements of motor performance and balance ability. The improvement of spatial working memory by the Y-maze test might be implicated by the hyperlocomotion which could significantly interfere with the measurement of spontaneous alternation^53^. Also, the forced swimming test has disadvantages on lacking sufficient mechanistic specificity, one-dimensional outcome and influencing behaviors by repeated trials. To specify and validate the behavioral phenotypes induced by the probiotics, further behavioral tests are required.

In this study, we observed stimulatory effects of JDFM216 immune system, such as enhanced phagocytic activity of macrophages and secreted intestinal sIgA. Beneficial effects of probiotics on the host intestinal immune functions have been identified, including modulation of innate immunity and priming of the humoral adaptive immune system^54,55^. Probiotics also play a role in maintaining the balance between innate and adaptive immune responses by directly interacting with intestinal epithelial cells or interacting with dendritic cells and follicle-associated epithelial cells via M cells, further initiating responses mediated by macrophages and lymphocytes^55^. These processes can be mediated by probiotics through inducing the production of different chemokines and modulating intestinal microbiota^56,57^. Our results support these current findings on the stimulatory effects of probiotics on the host immune modulation, however, we could not verify the specific cytokines expression affected by JDFM216 in the current study. We also measured the cortisol level in mice serum, but there was no significant difference between the two groups. Although the corticosterone is a main hormone involved in stress responses with an association of fear memory recognition, a recent study reported that circulating glucocorticoids are mostly bound to corticosteroid-binding globulin, and only approximately 5% of the hormones can cross the blood–brain barrier as free plasma glucocorticoids^58^. They also showed that intrahippocampal corticosterone generation was more important factor in stress-associated brain function than the blood corticosterone level. Therefore, it is still possible that the aged control mice would be more vulnerable to stress, although the blood corticosterone level was not significantly different to that of JDFM216-supplemented mice. In order to better understand the molecular mechanisms of JDFM216 that link the gut microbiota, immune, and central nervous systems in a network communication, further investigation on a broader range of immune responses will be conducted.

This study demonstrated the difference of gut microbiota composition between young and aged mice, and that treatment of JDFM216 was able to modulate the abundance of some OTUs including *Lactobacillus* itself. Previous studies have reported that administration of *Lactobacillus* strains modulates the gut microbiota and alleviates aging-induced metabolic diseases^59,60^. In this study, we observed that JDFM216 treatment significantly and mostly increased one OTU belonging to the genus *Lactobacillus,* the abundance of which was correlated to vitamin metabolism and other beneficial substances such as butanoate, lipoic acid, and taurine. Butanoate is probably the most reported beneficial metabolite produced by intestinal bacteria. It has been reported that consumption of conventional yogurt increases plasma vitamin B1, B2 and B6^61^, the metabolism of which was correlated to significantly increased *Lactobacillus* OTU by feeding with JDFM216. Lipoic acid is also known for its anti-aging properties through reducing oxidative stress^62^ and preventing memory loss^63^. Taurine is also known to increase hippocampal neurogenesis in aging mice^64^. These results suggest that such microbial production of functional metabolites may ultimately target diseases linked to aging and inflammation in host-microbial interactions.

The present study identifies associations of the gut microbiota, host immune system, and cognitive functions with respect to oxidative stress by increased phagocytic capacity, leukocyte respiratory burst activity, microbial metabolic products, and possibly altered cognitive behaviors. Oxidative stress has been implicated in a wide variety of degenerative processes including aging. It induces protein and DNA damage in conjunction with genetic instability and insufficient DNA damage repair^65^. Butanoate, lipoic acid, vitamin B6 and taurine, which are positively correlated with the OTU of *Lactobacillus*, are well known to have antioxidative effects by preventing oxygen radical generation^66^, antagonizing electrophilic oxidative stress^67^, and activating the transcription factor Nrf-2 (NF-E2 p45-related factor 2), which is a master regulator of cellular detoxification responses and redox status^17,68^. Recently, a number of studies have been published showing that probiotics have potent antioxidant capacity through the Nrf2 signaling pathway^69,70^. In addition, our previous studies revealed that the health-promoting effects of JDFM216 were mediated by a nuclear hormone receptor family and PMK-1 signaling cascade which regulates the oxidative stress response in *C. elegans*^44,48^. Further detailed mechanisms underlying gut bacteria-derived metabolite and aging-related antioxidant effects remain to be determined.

Taken together, administration of the probiotic *L. fermentum* strain JDFM216 in aged mice confirmed its features of the health promotion on the cognitive host responses and the immune system through the alteration of the intestinal taxonomic composition. The findings facilitate our understanding on the efficacy of probiotics and probiotic-derived factors on the regulation of the host psychological and immune health, thus providing a fascinating potential for probiotic application in human disease prevention and treatment.

## Methods

### Bacterial strains and growth conditions

*Lactobacillus fermentum* JDFM216, previously isolated from infant feces (referred to as JDFM216; Jang et al., 2017) was grown in Man, Rogosa and Sharpe (MRS) medium overnight at 37 °C. To prepare live bacterial feeding, strains were washed twice with sterile water and suspended in sterile water at a concentration of *ca* 1.0 × 10^9^ CFU/mL.

### Animals and experimental design

All mice were 6-week-old male C57BL/6 mice purchased from Orient Bio Inc. (Seongnam, Korea). Groups of 13–15 mice (randomly assigned to control or experimental groups) were housed in isolator cages (JD-C-07, Jeungdo Bio & Plant Co., Seoul, Korea) with sterilized bedding under the following auto-controlled conditions: lighting (12 h light and 12 h dark), temperature (24 °C), and relative humidity (55%). The acclimatization period was 2 weeks. Because C57BL/6 male mice have a long life span of approximately 26 to 28 months and are widely used for gerontological studies, we employed 12-month-old (52 weeks) mice of this strain as a model of middle-aged mice, and fecal and blood samples were first collected at 8 weeks for comparison with young mice (Young Groups). Figure 1A represents a schematic of the experimental plan. Initially, for the aging model of middle-aged mice, all mice maintained in sterile isolators and fed irradiated normal chow and water ad libitum for a total of 52 weeks. Then, sterile water was administered with or without living cells of JDFM216 (approximately 6.0 × 10^8^ CFU/mice/day) for 48 weeks to ensure that senescence was sufficiently developed (Aged Groups vs. Aged + JDFM216 Groups). At the indicated weeks (63, 77, and 100 weeks) during the administration period, fecal or blood samples were collected for analyzing fecal microbiota and biological features. To reduce stress during the long-term period of the experiment, all water administration was given freely, and the behavior tests were conducted sequentially from the least to the most stressful tasks. These tasks were designed for the measurement of spontaneous activity, short-term memory, motor function, locomotor activity, and depression level. All experiments were handled in accordance with institutional ethical guidelines, and this study was approved by the Institutional Animal Care and Use Committees (IACUC) of Jeonbuk National University (2015-05).

### Behavioral assessments

#### Y-maze test

For the measurement of learning and memory abilities, the Y-maze test was performed according to a method described previously^71^. The Y-maze apparatus consisted of a three-arm horizontal maze (35-cm-long and 5-cm-wide with 15-cm-high walls) in which the arms are symmetrically disposed at 120° angles from a central platform. Mice were initially placed at the end of one arm and allowed to explore freely. The number of arm entries serves as an indicator of locomotor activity, and its sequence was recorded manually for each mouse over an 8-m period. An actual alternation was defined as entries into all three arms for consecutive choices. The percentage of alternations was defined by the following equation: % Alternation = [(Number of actual alternations)/(Total arm entries − 2)] × 100.

#### Voluntary wheel-running test

All mice for the test were individually housed in cages containing running wheels. The running wheels consisted of a rotating metal chamber with a wire mesh floor (32 cm in diameter, 10 cm in width) attached to a stationary metal wall. Mice were trained for 20 min per day for three consecutive days inside a wheel. During the training session, the animal was free to run but could not leave the wheel. Distance (yards) of rotations was monitored for 300 s, and scores of individual profiles were averaged for each group.

#### Accelerated rotarod test

Evaluation of motor ability was performed using the accelerating treadmill rotarod instrument (BS Technolab) with automatic timers and falling sensors as described earlier^72^. The apparatus consisted of a black striated rod (3 cm in diameter) and 25 cm above the floor. Before the training sessions, the mice were habituated to stay on the stationary drum for 5 min. Habituation was repeated every day for 1 min immediately before the session. Mice were trained for one daily session consisting of three trials over three consecutive days. On the first day, the rotation speed was set at a fixed value of 10 rpm for 300 s. When an animal fell off during this period, it was placed back on the rod until the end of the trial. The speed for the second and third days was set to 20 and 30 rpm. After the last exposure, the rod was then set to accelerate from 4 to 40 rpm over a period of 300 s, and the time spent on the drum was recorded automatically for each mouse. Trials within the same day were performed with a 15-min rest period between trials. After testing was finished, the rod was cleaned and dried to ensure a non-slippery surface for the following animal. The data reported for each mouse are the average latency to fall from the rod for the three trials.

#### Balance beam test

This test measured the ability of the mice to traverse a progressive series of narrow wooden beam as described earlier^73^. The beam was made of wood and consisted of a square section that was 1 m in length and suspended horizontally 80 cm from the floor. Mice were trained over 2 days with three trials per day on the square beam (40 mm wide) until they could cross the beam quickly and without stopping. After training, each mouse was forced to walk along a narrow square beam (30 mm wide) to reach the end of the beam. The measurements taken were the number of foot slips and recorded with a maximum time of 300 s. The recorded data were scored as follows: 0, does not attempt to balance; 1, on the beam without moving; 2, turns on the left or right; 3, slipped over 50% while crossing; 4, slipped from two times to below 50% while crossing; 5, slipped only one time while crossing; and 6, crossed the beam without slips. For tests, scoring was carried out using video-tracking.

#### Forced swimming test (FST)

Depression behavior was assessed using the forced swim test as previously described^74^, which is a standard rodent test for screening antidepressant activity. The FST is one of the most used tools in preclinical research on depression treatment because it is a low-cost, fast, and reliable animal model with strong predictive validity. The experimental protocol requires mice to be placed in a water-filled large cylinder. The FST apparatus consisted of a transparent cylinder (10 cm diameter, 28 cm height) filled with water (18 cm, 24 ± 2 °C). During their first experience, mice were exposed to FST for 10 min (training). Twenty-four hours later, all the mice were exposed to the apparatus (test). The tests were assessed in a 6-min period, and the duration of immobility within the last 4 min of the test was recorded. The depression level is scored for immobile behavior, defined as stops struggling and floats motionless on the water, making only movements to maintain its head above water.

### Immunomodulatory assays

#### Detection of spleen indices

The body weight of mice was recorded, and then the mice were sacrificed by cervical dislocation. Spleens were obtained from the mice and weighed immediately. The spleen indices were measured as the ratio of the spleen weight to the mouse body weight.

#### Measurement of intestinal sIgA

After sacrificing the mice, intestinal samples from 5 cm of the cecum and distal sections of the intestine were collected and washed with PBS. The samples were homogenized in 0.5 mL PBS containing 1% protease inhibitor cocktail. The homogenized solution was centrifuged at 13,000 rpm for 15 min at 4 °C to remove particulate material. The supernatant was collected and assayed using commercially available ELISA kits for mouse secretory immunoglobulin A (sIgA) (Cat No. MBS269144, MyBioSource, San Diego, CA, USA) for sIgA determination.

#### Splenocyte proliferation assay

The splenocytes of each mouse groups were aseptically isolated using 70 μm cell strainer, washed and resuspended in ammonium chloride-Tris (ACT) buffer and deposited in 96-well plates at a concentration of 5.0 × 10^6^ cells/well with concanavalin A (ConA, 2 mg/mL). Splenocytes incubated with medium alone used as negative control. After cultivating Splenocytes and ConA for 96 h at 37 °C under atmosphere of 5% CO_2_, 20 µL of MTS solution (Promega, Madison, WI, USA) was treated. And then, the plate was incubated for additional 2 h and read at 490 nm (OD_490_) using a Synergy HTX Hybrid Multi Mode Microplate Reader (BioTek Instruments, Inc., Winooski, VT, USA). The proliferation of splenocyte were calculated by the stimulation index (SI): SI = OD_490_ of stimulated cells/OD_490_ of the negative control.

#### Determination of pinocytosis of peritoneal macrophage

In the present study, mice were sacrificed and peritoneal cells were cautiously collected by peritoneal lavage with PBS. Then, 3 mL of lavage fluid was harvested and centrifuged at 2000 rpm for 10 min. The collected cells were resuspended with the concentrations of 1 × 10^6^ cells/mL in RPMI1640 medium and located on 200 µL per well in 96-well plates. After culturing for 24 h at 37 °C, the medium was discarded, and then 100 µL of 0.072% neutral red solution was exposed to separate wells. Following additional cultivation for 30 min, each well was carefully washed three times with DPBS buffer. Next, 100 µL of lysis solution consisted of acetic acid and alcohol (1:1; v/v) was treated to each well, read at 570 nm (OD_570_) using microplate reader and finally evaluated as the phagocytosis index (PI). The PI was calculated according to the following formula: PI = (total number of engulfed cells/total number of counted macrophages) × (number of macrophages containing engulfed cells/total number of counted macrophages) × 100.

#### Serum analysis

Blood samples were collected after the mice were sacrificed. The fresh blood was kept standing for 60 min at 37 °C. Serum was obtained by centrifugation at 3000 rpm for 15 min and then stored at − 80 °C until use. Concentrations of cytokines including IL-4, IL-6, IL-10, CXCL1/KC, and TNF-α in serum were determined using a magnetic Luminex screening assay according to the manufacturer’s instructions (R&D Systems, Minneapolis, MN) on a Bio-Plex 200 analyzer (Bio-Rad, Hercules, CA). In addition, serum corticosterone level was measured using the corticosterone (Abnova, Taipei, Taiwan) and serotonin (Abcam, Cambridge, MA, USA) enzyme-linked immunosorbent assay kits, respectively, according to the manufacturer’s instructions.

#### CD11b^+^CD4^+^ lymphocyte subset analysis

The percentage of lymphocyte subpopulations in the spleen was measured using flow cytometric analysis. Approximately 9.0 × 10^7^ cells/mL of splenocytes were stained for CD4^+^ cell isolation using the MACS Treg isolation kit (Miltenyi Biotec, CA), and then they were separated using CD11b^+^ microbeads and a MACS LS column (Miltenyi Biotec, CA) according to the manufacturer’s directions. The mixed samples were washed twice with fluorescence-activated cell sorting (FACS) buffer (PBS, 1% FCS, and 0.09% sodium azide) and analyzed by flow cytometry (BD FACSCalibur). CD4^+^CD11b^+^ cells were expressed as proportion of cells positive for the markers per the number of total splenocytes.

#### Respiratory burst assay

The released amount of total O_2_^−^ from monocytes was determined as superoxide dismutase (SOD)-inhibitable reduction of JDFM216 at 570 nm in microtiter plates. The assay was carried out following a previous established method with modifications in the detection wavelength^75^. The method consists of a colorimetric determination of the ROS produced by the leukocyte respiratory burst, which promotes the reduction of nitroblue tetrazolium into a dark blue precipitate inside the phagocyte, called formazan granules. The cells were stimulated with PMA or PBS. Values in the absence of stimulus were subtracted from those obtained after stimulation.

### 16S rRNA sequencing metagenomic analysis

#### Fecal microbiota sequencing

Total DNA was extracted from 100 mg of feces collected from each mouse. The V4 region of the 16S ribosomal RNA gene was amplified by the polymerase chain reaction (PCR) using specific primers as previously described^76^. Following purification of the PCR products, individual indices were added to the amplicons by PCR. The amplicons were then sequenced using the Illumina MiSeq platform according to the manufacturer’s instructions. The replicates of the DNA samples were pooled and subjected to amplicon sequencing using MiSeq according to the manufacturer’s instructions. All sequencing was performed at Macrogen Inc. (Seoul, Republic of Korea).

#### Microbial community analysis

The bacterial composition and amplicon sequence analysis were conducted according to MiSeq SOP (https://www.mothur.org/wiki/MiSeq_SOP). Briefly, MOTHUR^77^ was used to assemble, align, and cluster the reads. Alignment was performed based on the database from SILVA (version 132)^78^, and taxonomic classification was performed based on the Ribosomal database project database^79^ and clustered based on OptiClust algorithms^80^ with a sequence distance 0.03. Chimeric sequences were removed using VSEARCH^81^. The distribution of operational taxonomic units (OTUs) was analyzed using STAMP^81^ based on the distance calculated using the Bray–Curtis coefficient. Gut microbiota derived metabolic activities were estimated using PICRUSt2 (https://github.com/picrust/picrust2). Functional pathways were annotated from Kyoto Encyclopedia of Genes and Genomes (KEGG, https://www.genome.jp) databases^82,83^.

#### Metabolites analysis

Culture supernatant of *L. fermentum* JDFM216 grown overnight in MRS were collected by centrifugation at 12,000 rpm for 10 min at 4 °C, followed by filtration through a 0.2-µm PVDF membrane. Extracellular metabolites were extracted by adding the culture supernatant to ice-cold methanol and vortexed for 1 min. After centrifugation at 10,000 rpm for 10 min at 4 °C, the upper layer of the supernatant was collected, concentrated to dryness in a vacuum concentrator, and stored at − 80 °C prior to derivatization and analysis by GC–MS.

The extract was derivatized with 60 µL of a solution of 20 mg/mL methoxyamine hydrochloride in pyridine (Sigma, St. Louis, MO, USA) at 30 °C for 90 min, and 100 µL of N,O-Bis(trimethylsilyl)trifluoroacetamide (BSTFA; Sigma) was subsequently added at 60 °C for 30 min. A mixture of fatty acid methyl esters and fluoranthene was added to the extract as internal standards. The GC–MS analysis was conducted using a Thermo Trace 1310 GC (Waltham, MA, USA) coupled to a Thermo ISQ LT single quadrupole mass spectrometer (Waltham, MA, USA). A DB-5MS column with 60-m length, 0.2-mm i.d. and 0.25-µm film thickness (Agilent, Santa Clara, CA, USA) was used for separation. For analysis, the sample was injected at 300 °C and split ratio 1:5 with 7.5 mL/min helium split flow. The metabolites were separated with 1.5 mL constant flow helium with an oven ramp of 50 °C (2 min hold) to 180 °C (8 min hold) at 5 °C/min, to 210 °C at 2.5 °C/min, and to 325 °C (10 min hold) at 5 °C/min. The mass spectra were acquired in a scan range of 35–650 m/z at an acquisition rate of 5 spectra per sec. The ionization mode was subjected to electron impact, and the temperature for the ion source was set to 270 °C. The spectra were processed by Thermo Xcalibur software (version 4.1, Waltham, MA, USA), using automated peak detection, and the metabolites were identified by matching the mass spectra and retention indices of the NIST Mass spectral search program (version 2.0, Gaithersburg, MD, USA).

### Statistics

Data were analyzed using GraphPad Prism software (version 8, San Diego, CA, USA). All results are presented as the mean ± standard deviation (SD). Statistical analysis was performed using the Student’s *t* test for comparisons between two groups and one-way analysis of variance for comparisons among multiple groups. *P* < 0.05 was considered significant. Spearman correlation analysis was applied to identify correlated OTUs to metabolic activities. Significant differences (*P* < 0.05) between samples were estimated at the OTU level.

## Supplementary information

Supplementary Information.

Supplementary Movie.
