# Supplementary material for: Probiotic Lactobacillus fermentum strain JDFM216 improves cognitive behavior and modulates immune response with gut microbiota
Source: Sci Rep. 2020 Dec 10;10:21701. doi: 10.1038/s41598-020-77587-w (PMC7729874; doi:10.1038/s41598-020-77587-w)
Supplement: Supplementary file 1 — Supplementary Information. [file 41598_2020_77587_MOESM1_ESM.docx]

**Supplementary Materials**

**Probiotic *Lactobacillus fermentum* strain JDFM216 improves cognitive behavior and modulates immune response with gut microbiota**

Mi Ri Park^1^, Minhye Shin^2^, Seong-Yeop Jeong^3^, Do-Youn Jeong^3^, Minho Song^4^, Gwangpyo Ko^5^, Tatsuya Unno^5,6^, Younghoon Kim^2*^, and Sangnam Oh^7*^

^1^Department of Animal Science and Institute of Milk Genomics, Chonbuk National University, Jeonju, 54896, Korea

^2^Department of Agricultural Biotechnology and Research Institute of Agriculture and Life Science, Seoul National University, Seoul 08826, Korea

^3^Microbial Institute for Fermentation Industry, Sunchang, Jeonbuk 56048, Korea

^4^Division of Animal and Dairy Science, Chungnam National University, Daejeon 34134, Korea

^5^Subtropical/tropical Organism Gene Bank, Jeju Natinoal University, Jeju 63243, Republic of Korea

^6^Faculty of Biotechnology, Jeju National University, Jeju 63243, Republic of Korea

^7^Department of Functional Food and Biotechnology, Jeonju University, Jeonju, 55069, Korea

^*^These authors contributed equally to this study

^*^Corresponding authors: Younghoon Kim and Sangnam Oh

E-mail: ykeys2584@snu.ac.kr and osangnam@jj.ac.kr

Phone: +82-63-220-3109, Fax: +82-63-220-2054

**
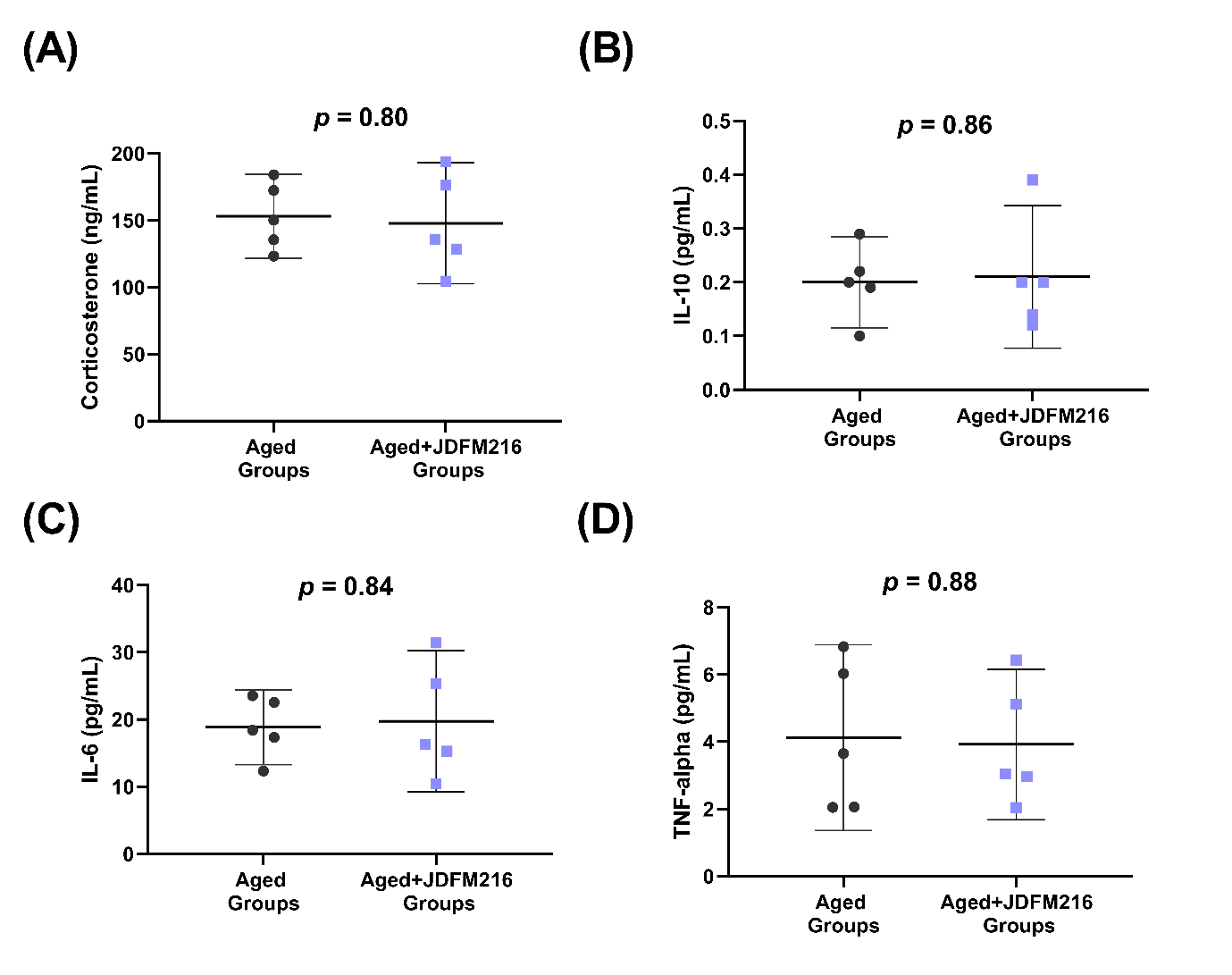
**

**Figure S1.** Profiling of the immune response in JDFM216-treated aged mice. (A) Corticosterone level of aged mice (Aged Groups or Aged+JDFM216 Groups). (B-D) Cytokines IL-10 (B), IL-6 (C) and TNF-alpha levels (D) of aged mice (Aged Groups or Aged+JDFM216 Groups). Values are means ± SD. Student’s *t* -test was performed and *p* values were indicated.

**
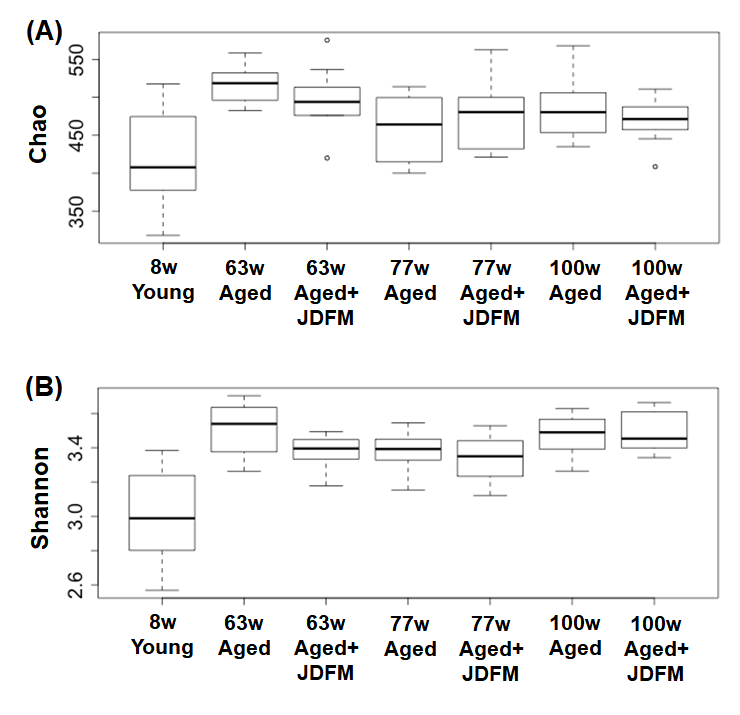
**

**Figure S2.** Comparison of ecological indices: (A) species richness and (B) species evenness estimated by Chao and Shannon indices, respectively.

**
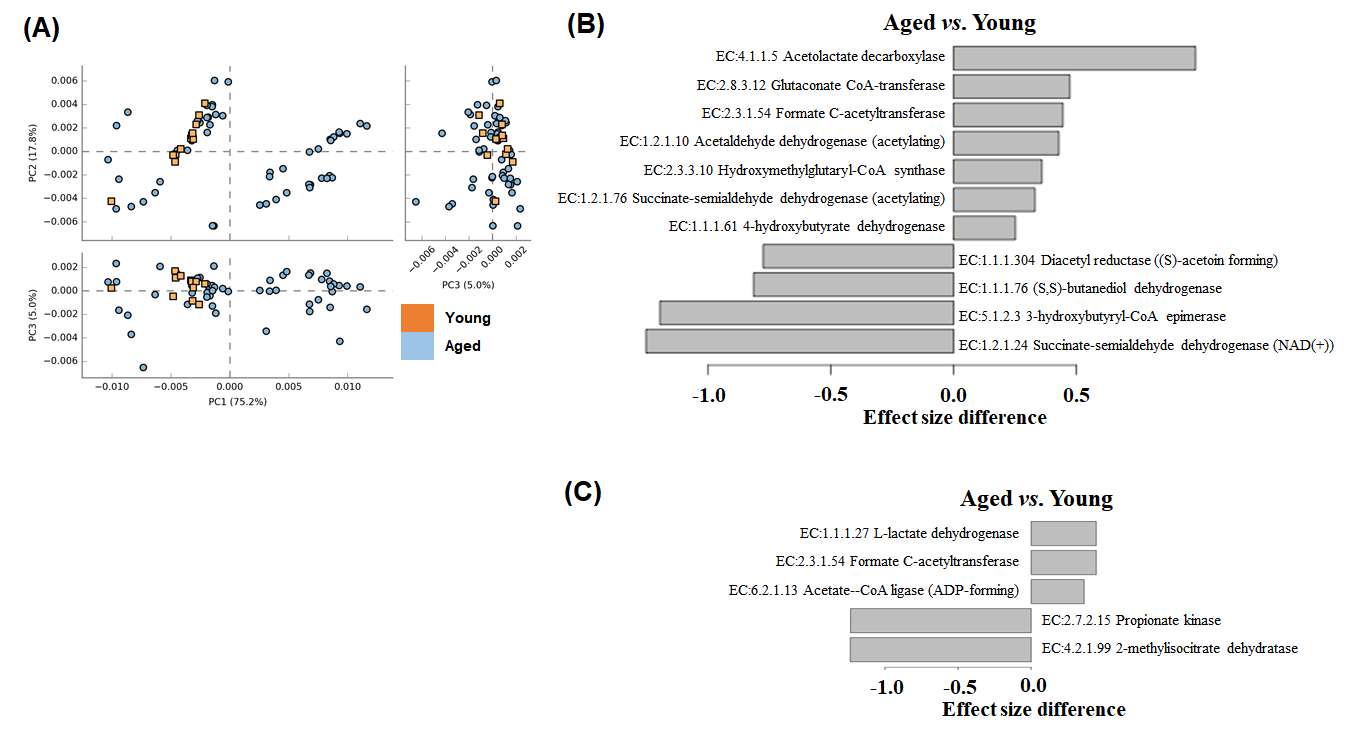
**

**Figure S3.** Comparison between Young Groups and Aged Groups for gut metabolic activities (A) and KEGG enzymes with significantly different abundance for butanoate (B) and propionate metabolisms (C).

**
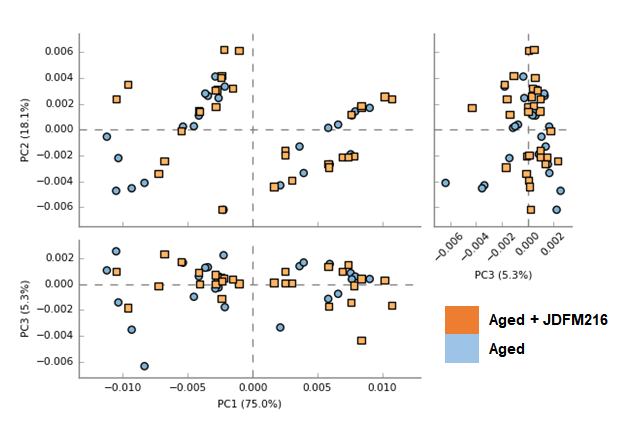
**

**Figure S4.** Comparison of metabolic activity profile differences between age mice (Aged Groups) and aged mice fed *Lactobacillus fermentum* JDFM216 mice (Aged Groups+JDFM216).


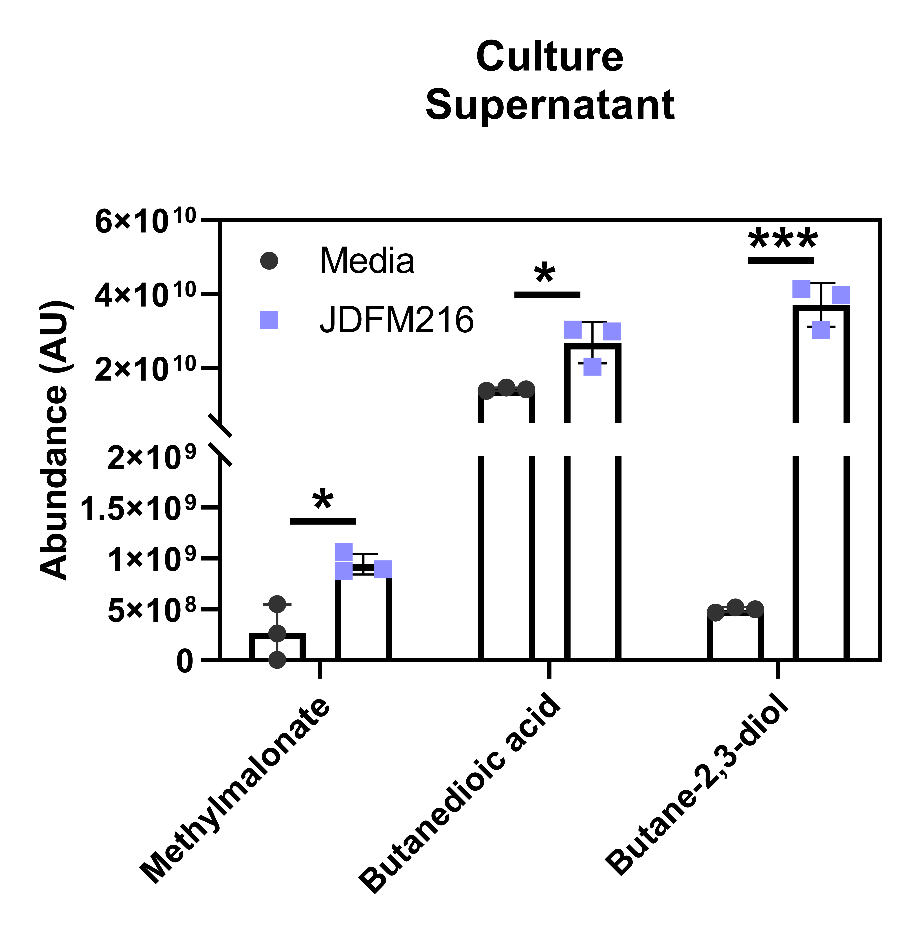


**Figure S5.** Comparison of butanoate-associated metabolites abundance between media and culture supernatant of *Lactobacillus fermentum* JDFM216. Metabolites abundance was expressed as an arbitrary unit (AU). Values are means ± SD. Asterisks represent statistically significant differences; Student’s t-test was performed. **p* < 0.05 and ****p* < 0.001, compared between the groups.

**Table S1.** Positively correlated KEGG enzymes with the abundance of OTU0023 *Lactobacillus*

| **EC #** | **Description** | **Involved Pathways** |
| --- | --- | --- |
| EC:1.1.1.337 | L-2-hydroxycarboxylate dehydrogenase (NAD(+)) | Methane metabolism |
| EC:1.1.1.350 | Ureidoglycolate dehydrogenase (NAD(+)) | Purine metabolism, Microbial metabolism in diverse environments |
| EC:1.1.1.388 | Glucose-6-phosphate dehydrogenase (NAD(+)) | Pentose phosphate pathway |
| EC:1.1.1.4 | (R,R)-butanediol dehydrogenase | Butanoate metabolism |
| EC:1.1.1.65 | Pyridoxine 4-dehydrogenase | Microbial metabolism in diverse environments, Vitamin B6 metabolism |
| EC:1.18.6.1 | Nitrogenase | Chloroalkane and chloroalkene degradation, Microbial metabolism in diverse environments, Nitrogen metabolism |
| EC:1.4.3.19 | Glycine oxidase | Thiamine metabolism |
| EC:2.1.1.184 | 23S rRNA (adenine(2085)-N(6))-dimethyltransferase | NA |
| EC:2.1.1.185 | 23S rRNA (guanosine(2251)-2'-O)-methyltransferase | NA |
| EC:2.3.1.109 | Arginine N-succinyltransferase | Arginine and proline metabolism |
| EC:2.3.1.16 | Acetyl-CoA C-acyltransferase | alpha-Linolenic acid metabolism, Benzoate degradation, Biosynthesis of antibiotics, Biosynthesis of secondary metabolites, Biosynthesis of unsaturated fatty acids,  Ethylbenzene degradation, Fatty acid degradation, Fatty acid elongation, Geraniol degradation, Microbial metabolism in diverse environments, Valine, leucine and isoleucine degradation |
| EC:2.3.1.200 | Lipoyl amidotransferase | Lipoic acid metabolism |
| EC:2.3.1.57 | Diamine N-acetyltransferase | Arginine and proline metabolism |
| EC:2.3.2.2 | Gamma-glutamyltransferase | Cyano amino acid metabolism, Glutathione metabolism, Taurine and hypo taurine metabolism |
| EC:2.3.3.15 | Sulfoacetaldehyde acetyltransferase | Taurine and hypo taurine metabolism |
| EC:2.4.1.18 | 1,4-alpha-glucan branching enzyme | Biosynthesis of secondary metabolites, Starch and sucrose metabolism |
| EC:2.4.99.16 | Starch synthase (maltosyl-transferring) | Starch and sucrose metabolism |
| EC:2.5.1.46 | Deoxyhypusine synthase | NA |
| EC:2.5.1.49 | O-acetylhomoserine aminocarboxypropyltransferase | Cysteine and methionine metabolism |
| EC:2.7.1.35 | Pyridoxal kinase | Vitamin B6 metabolism |
| EC:2.7.1.48 | Uridine kinase | Drug metabolism-other enzymes, Pyrimidine metabolism |
| EC:2.7.4.6 | Nucleoside-diphosphate kinase | Biosynthesis of antibiotics, Biosynthesis of secondary metabolites, Drug metabolism-other enzymes, Purine metabolism, Pyrimidine metabolism |
| EC:2.7.7.12 | UDP-glucose--hexose-1-phosphate uridylyltransferase | Amino sugar and nucleotide sugar metabolism, Galactose metabolism |
| EC:3.2.1.25 | Beta-mannosidase | Other glycan degradation |
| EC:3.4.23.36 | Signal peptidase II | NA |
| EC:3.5.2.14 | N-methylhydantoinase (ATP-hydrolyzing) | Arginine and proline metabolism |
| EC:4.1.99.12 | 3,4-dihydroxy-2-butanone-4-phosphate synthase | Biosynthesis of secondary metabolites, Biosynthesis of secondary metabolites, Riboflavin metabolism |
| EC:5.1.3.30 | D-psicose 3-epimerase | NA |

**Table S2.** Negatively correlated KEGG enzymes with the abundance of OTU0023 *Lactobacillus*

| **EC #** | **Description** | **Involved Pathways** |
| --- | --- | --- |
| EC:1.13.11.53 | Acireductone dioxygenase (Ni(2+)-requiring) | Cysteine and methionine metabolism |
| EC:1.14.19.2 | Stearoyl-[acyl-carrier-protein] 9-desaturase | Fatty acid biosynthesis, Biosynthesis of unsaturated fatty acids |
| EC:1.14.99.48 | Heme oxygenase (staphylobilin-producing) | Porphyrin and chlorophyll metabolism, Biosynthesis of secondary metabolites |
| EC:1.2.1.16 | Succinate-semialdehyde dehydrogenase (NAD(P)(+)) | Alanine, aspartate and glutamate metabolism, Tyrosine metabolism, Butanoate metabolism, Nicotinate and nicotinamide metabolism, Microbial metabolism in diverse environments |
| EC:1.2.7.6 | Glyceraldehyde-3-phosphate dehydrogenase (ferredoxin) | Glycolysis / Gluconeogenesis, Microbial metabolism in diverse environments |
| EC:1.20.4.1 | Arsenate reductase (glutaredoxin) | NA |
| EC:1.3.1.9 | Enoyl-[acyl-carrier-protein] reductase (NADH) | Fatty acid biosynthesis |
| EC:1.3.99.31 | Phytoene desaturase (lycopene-forming) | Carotenoid biosynthesis, Biosynthesis of secondary metabolites |
| EC:1.4.3.3 | D-amino-acid oxidase | Glycine, serine and threonine metabolism, Penicillin and cephalosporin biosynthesis, Arginine and proline metabolism, D-Arginine and D-ornithine metabolism, Biosynthesis of antibiotics |
| EC:2.1.1.250 | [Trimethylamine--corrinoid protein] Co-methyltransferase | Methane metabolism, Microbial metabolism in diverse environments |
| EC:2.1.3.12 | Decarbamoylnovobiocin carbamoyltransferase | Novobiocin biosynthesis, Biosynthesis of secondary metabolites, Biosynthesis of antibiotics |
| EC:2.3.1.101 | Formylmethanofuran--tetrahydromethanopterin N-formyltransferase | Methane metabolism, Microbial metabolism in diverse environments |
| EC:2.3.1.30 | Serine O-acetyltransferase | Cysteine and methionine metabolism, Sulfur metabolism, Biosynthesis of various secondary metabolites - part 3, Biosynthesis of secondary metabolites, Microbial metabolism in diverse environments, Biosynthesis of antibiotics |
| EC:2.3.1.89 | Tetrahydrodipicolinate N-acetyltransferase | Lysine biosynthesis, Metabolic pathways, Biosynthesis of secondary metabolites |
| EC:2.4.1.310 | Vancomycin aglycone glucosyltransferase | Biosynthesis of vancomycin group antibiotics |
| EC:2.5.1.43 | Nicotianamine synthase | NA |
| EC:2.6.1.57 | Aromatic-amino-acid transaminase | Cysteine and methionine metabolism, Lysine biosynthesis, Tyrosine metabolism, Phenylalanine metabolism, Phenylalanine, tyrosine and tryptophan biosynthesis, Novobiocin biosynthesis, Isoquinoline alkaloid biosynthesis, Tropane, piperidine and pyridine alkaloid biosynthesis, Biosynthesis of secondary metabolites, Biosynthesis of antibiotics |
| EC:2.7.7.72 | CCA tRNA nucleotidyltransferase | NA |
| EC:2.8.3.21 | L-carnitine CoA-transferase | NA |
| EC:3.1.1.81 | Quorum-quenching N-acyl-homoserine lactonase | NA |
| EC:3.1.3.12 | Trehalose-phosphatase | Starch and sucrose metabolism, Biosynthesis of secondary metabolites |
| EC:3.1.3.4 | Phosphatidate phosphatase | Glycerolipid metabolism, Glycerophospholipid metabolism, Ether lipid metabolism, Sphingolipid metabolism, Biosynthesis of secondary metabolites |
| EC:3.4.21.96 | Lactocepin | NA |
| EC:3.5.1.11 | Penicillin amidase | Penicillin and cephalosporin biosynthesis, Biosynthesis of antibiotics |
| EC:3.5.1.56 | N,N-dimethylformamidase | Glyoxylate and dicarboxylate metabolism |
| EC:3.6.1.11 | Exopolyphosphatase | Purine metabolism |
| EC:4.1.1.83 | 4-hydroxyphenylacetate decarboxylase | NA |
