# Supplementary material for: Probiotic Lactobacillus fermentum strain JDFM216 improves cognitive behavior and modulates immune response with gut microbiota
Source: Sci Rep. 2020 Dec 10;10:21701. doi: 10.1038/s41598-020-77587-w (PMC7729874; doi:10.1038/s41598-020-77587-w)
Supplement: Supplementary file 2 — Supplementary Movie. [file 41598_2020_77587_MOESM2_ESM.pptx]

## Slide 1
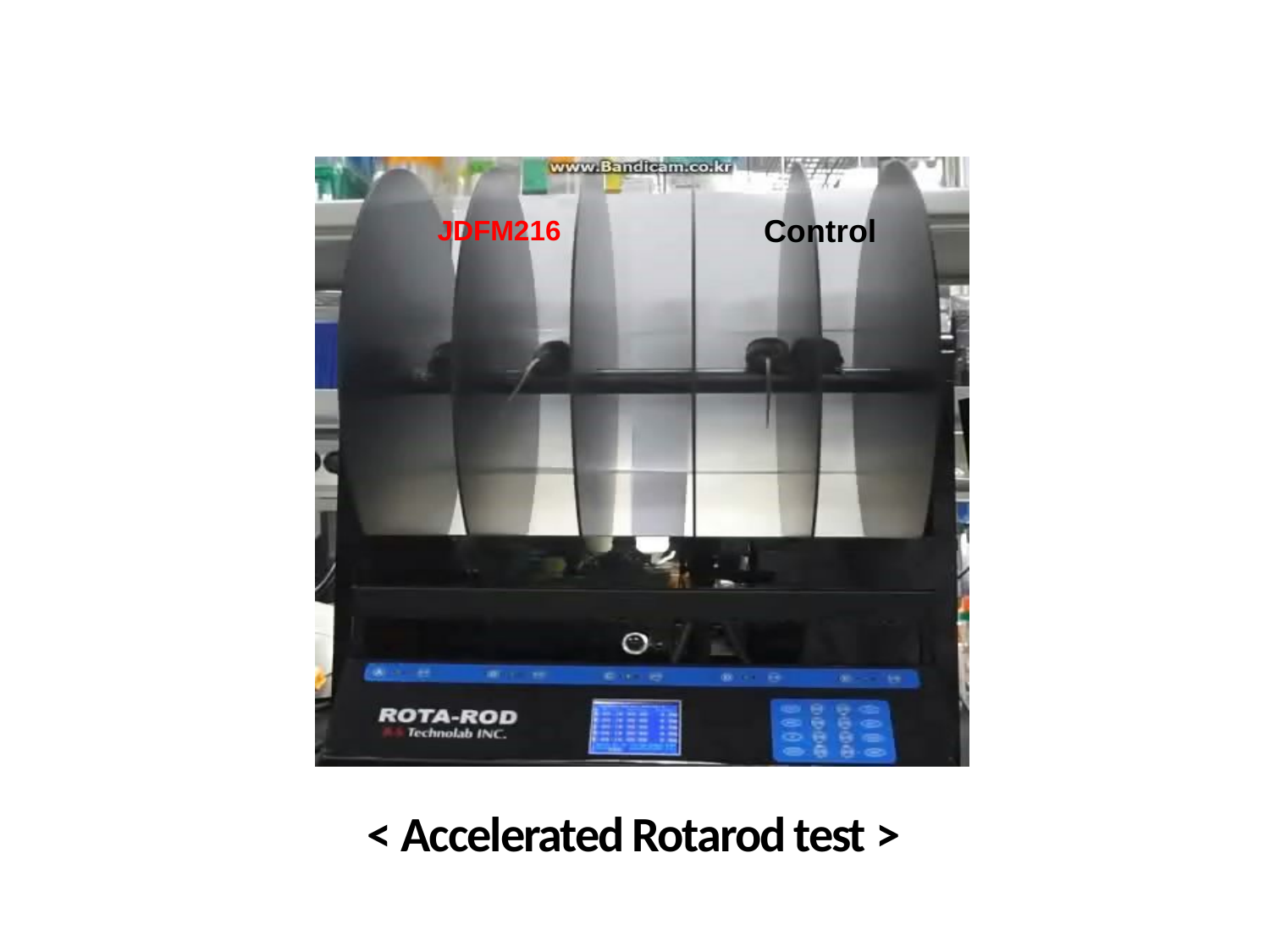

Control
JDFM216
< Accelerated Rotarod test >

## Slide 2
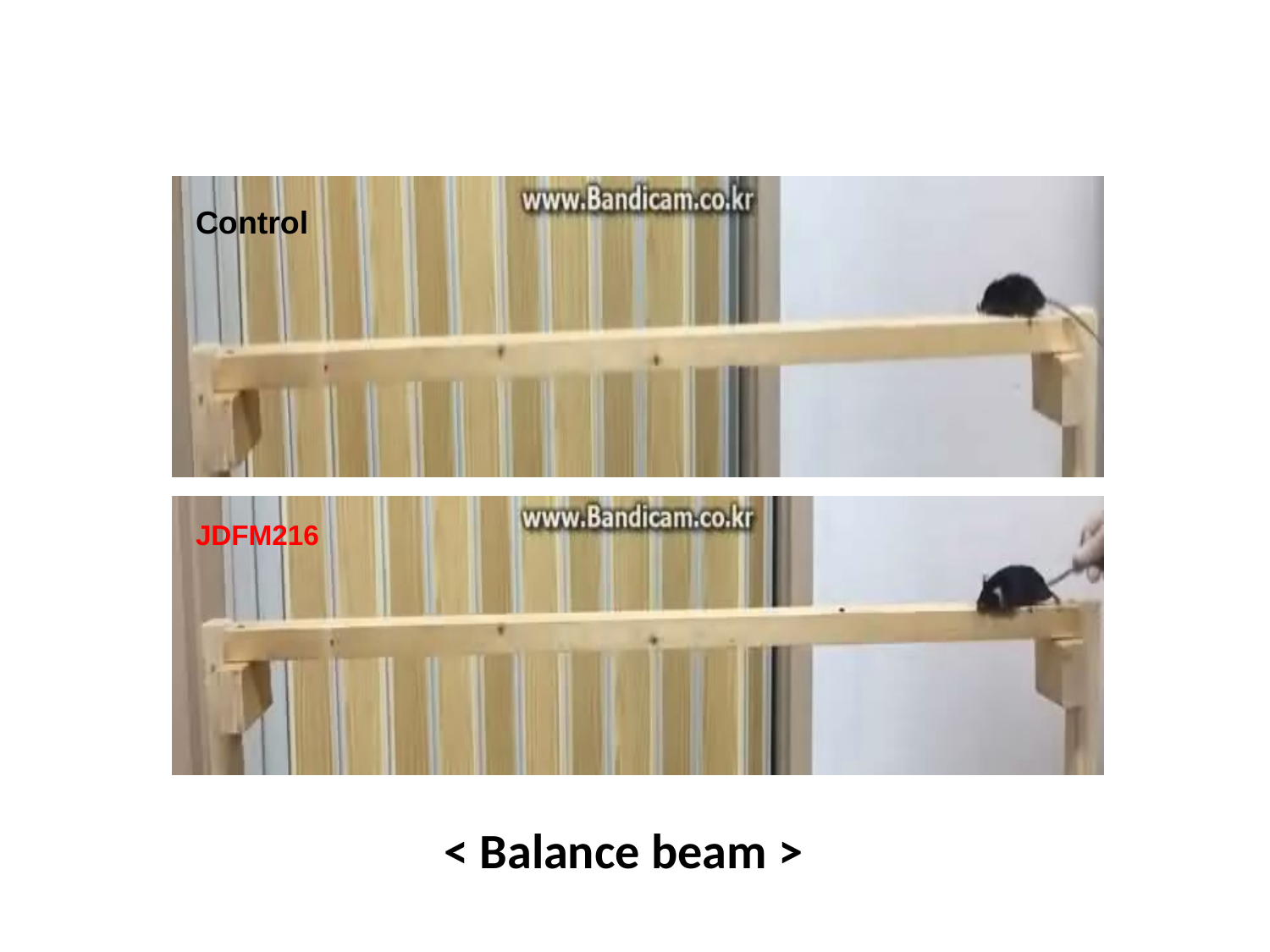

Control
JDFM216
< Balance beam >

## Slide 3
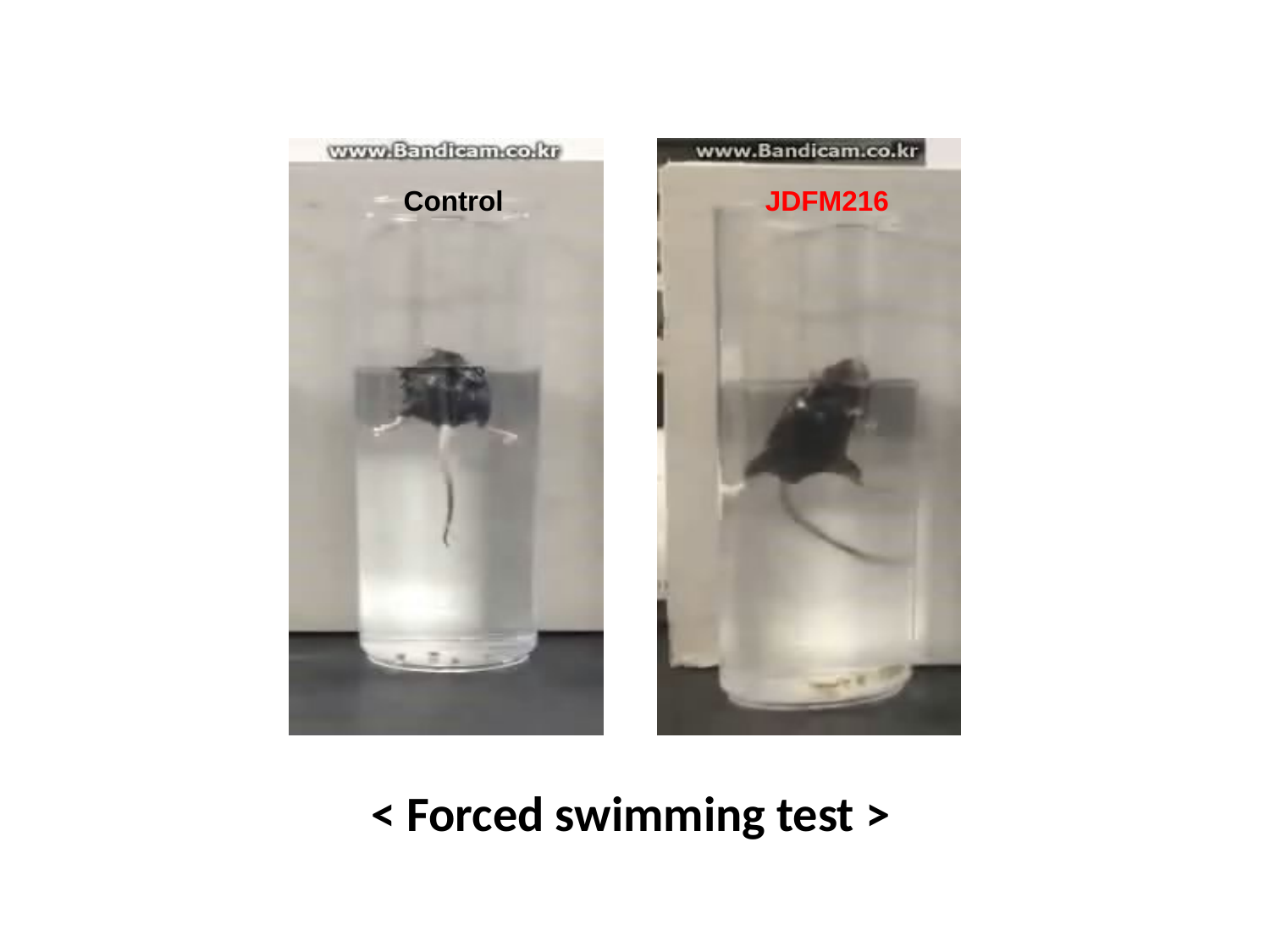

Control
JDFM216
< Forced swimming test >
